# Cycloserine Population Pharmacokinetics and Pharmacodynamics in Patients with Tuberculosis

**DOI:** 10.1128/AAC.00055-19

**Published:** 2019-04-25

**Authors:** Wael A. Alghamdi, Abdullah Alsultan, Mohammad H. Al-Shaer, Guohua An, Shahriar Ahmed, Yosra Alkabab, Sayera Banu, Ketevan Barbakadze, Eric Houpt, Maia Kipiani, Lali Mikiashvili, Stephan Schmidt, Scott K. Heysell, Russell R. Kempker, J. Peter Cegielski, Charles A. Peloquin

**Affiliations:** a Department of Pharmacotherapy and Translational Research, College of Pharmacy, University of Florida, Gainesville, Florida, USA; b Department of Clinical Pharmacy, College of Pharmacy, King Khalid University, Abha, Saudi Arabia; c Department of Clinical Pharmacy, College of Pharmacy, King Saud University, Riyadh, Saudi Arabia; d Division of Pharmaceutics and Translational Therapeutics, College of Pharmacy, University of Iowa, Iowa City, Iowa, USA; e Infectious Diseases Division, International Centre for Diarrhoeal Diseases Research, Bangladesh (icddr,b), Dhaka, Bangladesh; f Division of Infectious Diseases and International Health, Department of Medicine, University of Virginia, Charlottesville, Virginia, USA; g National Center for TB and Lung Diseases (NCTLD), Tbilisi, Georgia; h Department of Pharmaceutics, College of Pharmacy, University of Florida, Orlando, Florida, USA; i Division of Infectious Diseases, Department of Medicine, Emory University, Atlanta, Georgia, USA; j University of Texas Health Science Center at Tyler, Tyler, Texas, USA

**Keywords:** cycloserine, drug-resistant tuberculosis, pharmacodynamics, pharmacokinetics, target attainment

## Abstract

Limited pharmacokinetic/pharmacodynamic (PK/PD) data exist on cycloserine in tuberculosis (TB) patients. We pooled several studies into a large PK data set to estimate the population PK parameters for cycloserine in TB patients. We also performed simulations to provide insight into optimizing the dosing of cycloserine. TB patients were included from Georgia, Bangladesh, and four U.S. sites. Monolix and mlxR package were used for population PK modeling and simulation. We used PK/PD targets for time above MIC of ≥30% and ≥64%, representing bactericidal activity and 80% of the maximum kill, to calculate the probability of target attainment (PTA). Optimal PK/PD breakpoints were defined as the highest MIC to achieve ≥90% of PTA. Data from 247 subjects, including 205 patients with drug-resistant TB, were included. The data were best described by a one-compartment model. In most cases, the PK/PD breakpoints for the simulated regimens were similar for both PK/PD targets. Higher PTA were achieved as the total daily dose was increased. The highest PK/PD breakpoint that resulted from the use of 250 mg dosages was 16 mg/liter. For MICs of >16 mg/liter, doses of at least 500 mg three times daily or 750 mg twice daily were needed. In conclusion, the current dosing for cycloserine, 250 to 500 mg once or twice daily, is not sufficient for MICs of >16mg/liter. Further studies are needed regarding the efficacy and tolerability of daily doses of >1,000 mg. Dividing the dose minimally affected the PK/PD breakpoints while optimizing exposure, which can potentially reduce adverse drug effects.

## Abstract (precis)

Limited pharmacokinetic/pharmacodynamic (PK/PD) data exist on cycloserine in tuberculosis (TB) patients. We pooled several studies into a large PK data set to estimate the population PK parameters for cycloserine in TB patients.

## INTRODUCTION

Tuberculosis (TB) continues to claim millions of lives annually and is the leading cause of death from a single infectious agent (1). When Mycobacterium tuberculosis develops resistance to rifampin and isoniazid and is known as multidrug-resistant TB (MDR-TB), treatment requires the use of second-line drugs (SLDs), which are less effective and more toxic than regimens used for drug-susceptible TB (DS-TB). Optimized dosing of current SLDs offers one way to improve existing therapy for MDR-TB while waiting for newer and more effective agents to become available. Cycloserine, a cyclic analogue of d-alanine, is an SLD for TB that was discovered in 1954 (2). It competitively inhibits alanine racemase and d-alanine d-alanine ligase, two key sequential enzymes needed for *M. tuberculosis* cell wall synthesis (3). Recently, the World Health Organization (WHO) has reclassified cycloserine and now recommends its use as part of the regimen for all MDR-TB patients who do not qualify for the shorter MDR-TB regimen (4).

Recent work by Yu et al. found that a ratio of cycloserine peak serum concentration to MIC (maximum [peak] concentration [*C*_max_]/MIC) of ≥1 was associated with favorable outcomes (5). However, they measured a single concentration at 2 hours and did not explore other pharmacodynamic (PD) indices. In hollow-fiber systems, Deshpande et al. have recently demonstrated that time above MIC (*T*_>MIC_) is the key driver for cycloserine efficacy (6), a previously hypothesized PD index owing to its inhibition of the bacteria peptidoglycan synthesis, similar to that of the beta-lactams. Cycloserine also binds to *N*-methyl-d-aspartate receptors, which in part explains the commonly associated neurotoxicity and also, relatedly, has led to research into its use for psychiatric indications at lower doses (7, 8). At currently recommended anti-TB dosing for cycloserine (250 to 500 mg once or twice daily), the neurotoxicity can range from mild to severe and has resulted in psychosis and treatment discontinuation in some cases (9–12). These adverse events are thought to be associated with elevated cycloserine plasma concentrations, although no study has examined this relationship.

Despite the introduction of cycloserine over half a century ago, there are limited pharmacokinetic/pharmacodynamic (PK/PD) data on cycloserine in TB patients. In our study, we pooled relatively large PK data sets for cycloserine from several studies to estimate population PK parameters in TB patients, mainly MDR-TB, and explored covariates that might contribute to the variability of drug exposure. We also performed Monte Carlo simulations and target attainment analyses to better understand the optimal dosing of cycloserine and whether the current recommended doses are sufficient.

## RESULTS

### Population demographics.

A total of 235 TB patients and 12 healthy subjects were included in the model. The median (interquartile range [IQR]) age and weight were 41.0 (28.9 to 52.0) years and 59.0 (51.4 to 68.6) kg, respectively. Approximately 75% of the patients were males. Over 80% of the included subjects had MDR, preextensively drug-resistant (pre-XDR), or XDR-TB (Table 1).

**TABLE 1:** Demographic data for subjects included in the population pharmacokinetic model

Characteristic^*a*^	Median (IQR) or no. (%) for:	
	Healthy subjects (*n* = 12)	Patients (*n* = 235)
Age (yrs)	36.1 (27.9–43.9)	41.0 (29.0–52.7)
Sex, male	6 (50.0)	179 (76.2)
Wt (kg)	77.3 (74.9–83.2)	58.0 (50.6–67.0)
BMI (kg/m^2^)	25.7 (23.0–28.2)	20.4 (18.4–22.9)
Patient diagnosis
NTM		14 (6.0)
DS-TB		16 (6.8)
RR/MDR-TB		160 (68.1)
Pre-XDR-TB		36 (15.3)
XDR-TB		9 (3.8)
SrCr (mg/dl)	0.90 (0.73–1.00)	0.90 (0.70–1.03)
CrCL (ml/min)	108.8 (98.9–139.9)	89.1 (68.8–111.9)

a BMI, body mass index; CrCL, creatinine clearance; DS, drug susceptible; NTM, nontuberculous mycobacteria; pre-XDR, preextensively drug-resistant; RR/MDR, rifampin resistant/multidrug resistant; SrCr, serum creatinine; TB, tuberculosis; XDR, extensively drug-resistant.

### Population pharmacokinetic analysis.

**FIG 1:**
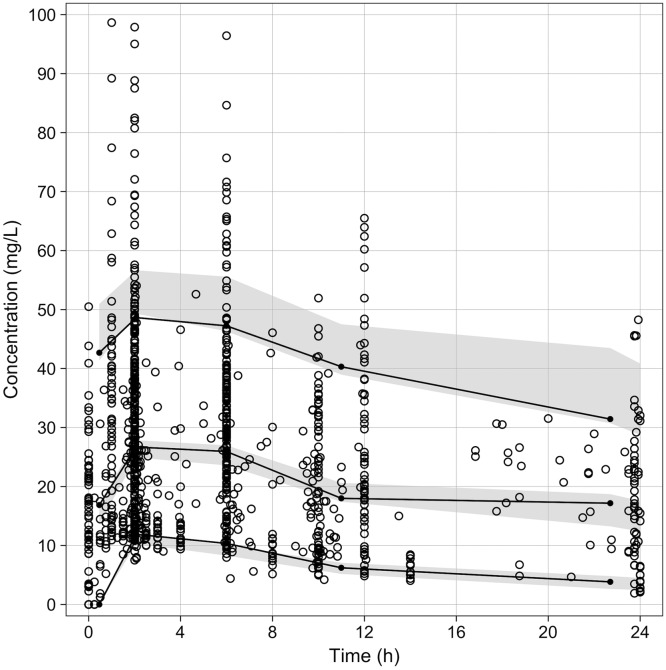
Visual predictive checks. Observed cycloserine concentrations are shown as open circles. Solid lines are the 5th, 50th, and 95th percentiles of the observed concentrations. The gray areas represent the 95% confidence intervals of the 5th, 50th, and 95th percentiles of the simulated cycloserine concentrations.

The number of cycloserine plasma concentrations used in the PK model was 1,069. The median (range) cycloserine peak concentration was 26.5 (7.5 to 97.9) mg/liter. Six patients, five from Bangladesh and one from Georgia, had a *C*_max_ of ≥80 mg/liter due to their high average dose (13.1 mg/kg) compared to the average dose (7.7 mg/kg) given to the rest of the population. The structural model was based on data from the intensively sampled healthy subjects. The remaining data sets that contained semirich and sparse data from patients with TB were added next. The entire data set was best described by a one-compartment model, with a first-order absorption and lag phase. Adding a parameter for a lag time resulted in a better fit during the absorption phase (Δ −2 × log-likelihood [−2LL] = –1,035.1). The proportional model was selected to estimate the residual error. The addition of weight on the apparent volume of distribution (*V*/*F*) followed allometric scaling, with the exponent fixed to 1. Of the other covariates evaluated, the presence or absence of disease (healthy subjects versus patients with TB) and creatinine clearance (CrCL) had significant effects on the apparent clearance (CL/*F*), while body weight had a significant impact on *V*/*F*. The difference in −2LL from the base and final models was –60 (Table 2). When those covariates were added in the final model, the interindividual variabilities decreased from 0.49 to 0.35 in CL/*F* and from 0.24 to 0.17 in *V*/*F*. The CL/*F* of cycloserine was estimated to be 2.00 liter/h in healthy subjects and 1.03 liter/h in patients, while *V*/*F* was estimated to be 24.9 liters. The estimated population PK parameters are presented in Table 2. The observations versus individual and population predictions are shown in Fig. S1A and B. The individual and population-weighted residuals versus concentrations are shown in Fig. S1C and D. Figure 1 shows the visual predictive check (VPC) for the entire data set; further stratifications by dose are shown in Fig. S2. For a 250-mg dose, there was a variety of dosing frequencies, including irregular twice daily dosing (e.g., doses at time 0 and 6 hours).

**TABLE 2:** Estimated population PK parameters in the base and final models

Parameter^*a*^	Parameter estimate (RSE [%])^*b*^		*P* value
	Base model	Final model	
−2LL	4,926.1	4,866.1	
Fixed-effect parameters
*T*_lag_ (h)	0.333 (10.6)	0.326 (1.47)	
*k_a_* (h^−1^)	7.25 (34.4)	6.61 (17.1)	
*V*/*F* (liter)	28.5 (4.05)	24.9 (2.92)	
β_V, wt_		1.00, fixed	
CL/*F* (liter/h)	1.02 (3.58)	2.00 (11.9)	
β_CL, patients (vs HS)_		−0.660 (18.7)	<0.0001
β_CL, CrCL_		0.413 (18.1)	<0.0001
Random-effect parameters
ω, *T*_lag_	0.368 (61.5)	0.409 (22.5)	
ω, *k_a_*	1.08 (19.2)	1.52 (13.6)	
ω, *V*/*F*	0.242 (16.7)	0.174 (36.6)	
ω, CL/*F*	0.492 (5.59)	0.353 (9.29)	
γ, CL/*F*		0.190 (21.1)	
Residual error parameter
Proportional	0.202 (3.04)	0.190 (3.37)	

a −2LL, −2 × log-likelihood; β, the estimated effect of the covariate; γ, interoccasion variability; ω, between-subject variability; CL/*F*, apparent clearance; CrCL, creatinine clearance; HS, healthy subjects; *k_a_*, absorption rate constant; SE, standard error; *T*_lag_, lag time; *V*/*F*, apparent volume of distribution; wt, body weight.

b RSE, relative standard error.

### Monte Carlo simulations.

**FIG 2:**
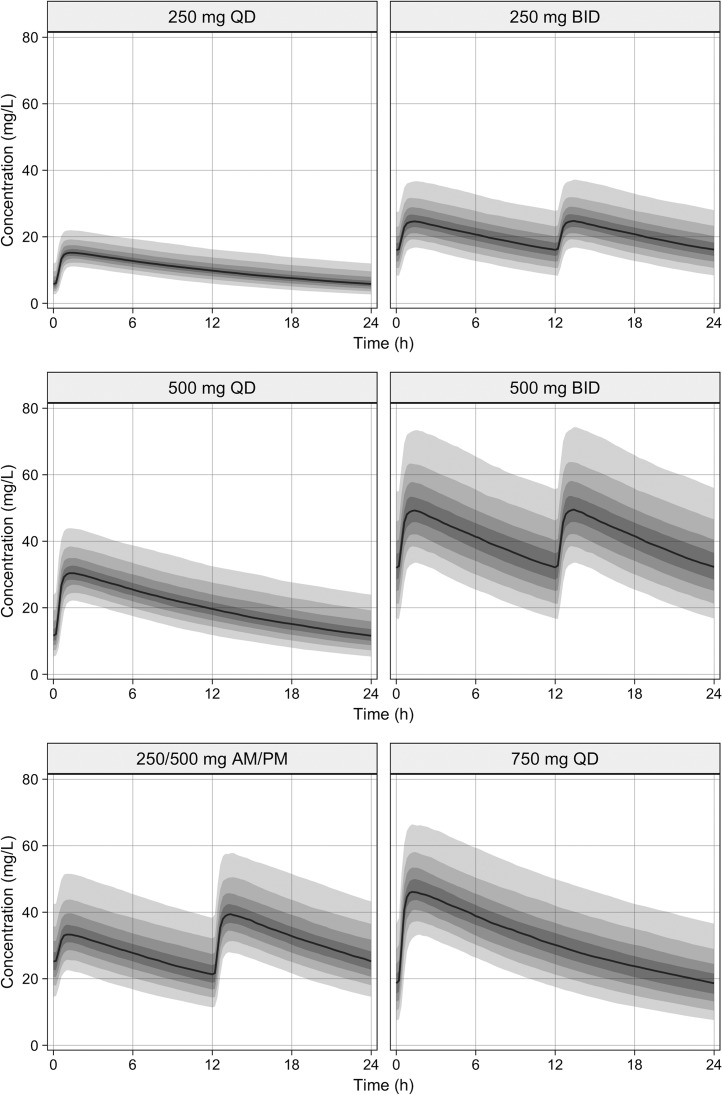
The empirical distribution of the simulated data for the most commonly used dosage regimens. The black line represents the median. The shaded area represents the 90% interval. The degree of the shaded area changes every 10th percentile.

**FIG 3:**
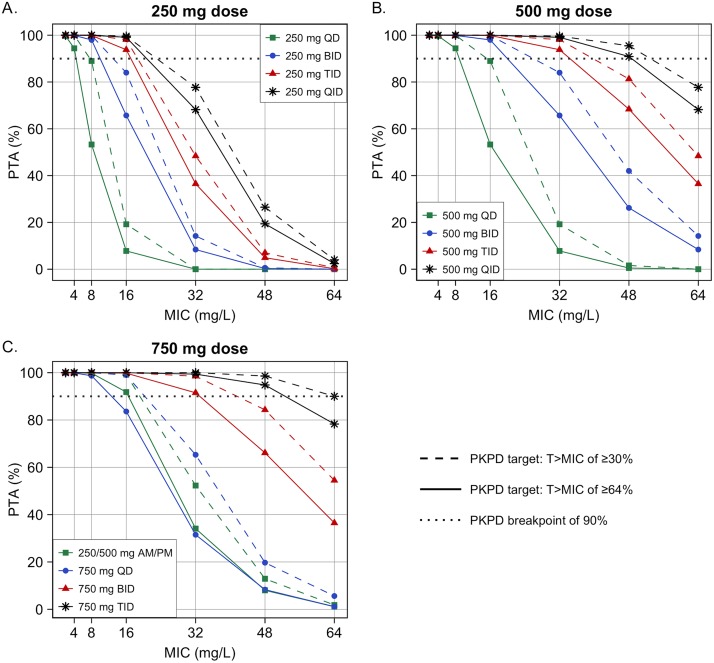
Probability of target attainment for the simulated cycloserine dosage regimens. Dosage regimens using a (A) 250-mg dose, (B) 500-mg dose, and (C) 750-mg dose.

The empirical distribution of the simulated data for the most commonly used dosage regimens is shown in Fig. 2. The PK/PD breakpoints for the simulated regimens were similar for both *T*_>MIC_ targets, except for the 750 mg once daily and three times daily regimens (Table 3). As the total daily dose of cycloserine was increased, the probability of target attainment (PTA) also increased (i.e., 250 mg versus 500 mg versus 750 mg given once daily). The 250 mg dosage regimens failed to achieve the prespecified PTA for MICs of >16 mg/liter (Fig. 3). MICs of 32 and 48 mg/liter required at least 500 mg three times daily and 750 mg three times daily, respectively, to achieve ≥90% of the PTA.

**TABLE 3:** The PK/PD breakpoints, *C*_max_, and AUC_0–24h_ for the simulated dosage regimens

Dosage regimen^*b*^	PK/PD breakpoint^*a*^(mg/liter)		Mean (SD)	
	*T*_>MIC_ ≥ 30%	*T*_>MIC_ ≥ 64%	*C*_max_ (mg/liter)	AUC_0–24h_ (mg · h/liter)
250-mg dose				
Once daily	4	4	16.4 (4.3)	259.5 (97.9)
Twice daily	8	8	26.4 (8.0)	516.7 (188.3)
Three times daily	16	16	35.5 (10.4)	737.7 (239.1)
Four times daily	16	16	44.4 (12.5)	945.1 (279.8)
500-mg dose				
Once daily	8	8	32.7 (8.6)	519.0 (195.7)
Twice daily	16	16	52.9 (16.0)	1,033.4 (376.7)
Three times daily	32	32	71.0 (20.7)	1,475.4 (478.3)
Four times daily	48	48	88.8 (24.9)	1,890.1 (559.6)
750-mg dose				
Split to 250/500 mg (a.m./p.m.)	16	16	42.2 (11.7)	763.8 (271.0)
Once daily	16	8	49.6 (13.2)	789.5 (304.6)
Twice daily	32	32	78.4 (22.9)	1,527.5 (537.3)
Three times daily	64	48	106.5 (30.6)	2,215.0 (702.6)

a PK/PD breakpoint defined as the highest MIC where at least 90% of the PTA was achieved. PK/PD, pharmacokinetic(s)/pharmacodynamic(s).

b AUC_0–24h_, area under the drug concentration-time curve from time 0 to 24 hours; *C*_max_, maximum (peak) concentration; PTA, probability of target attainment; *T*_>MIC_, time above the MIC.

Dividing the daily dose did not improve the PK/PD breakpoints, due to the relatively long half-life of 16.8 h for cycloserine. An exception to that was dividing the 750 mg dose into 250/500 mg daily, which resulted in a PK/PD breakpoint of 16 mg/liter, compared to 8 mg/liter in the 750 mg once daily regimen. Assuming that *C*_max_ is the predictor for drug-associated neuropsychiatric toxicity, dividing the daily dose reduced the *C*_max_ significantly (Table 3 and Fig. 2). For example, the *C*_max_ values for 500 mg once daily and 250 mg twice daily were 33 and 26 mg/liter, respectively. Similarly, the *C*_max_ values for 750 mg once daily and the 250/500 mg regimen were 50 and 42 mg/liter, respectively.

## DISCUSSION

In our model, we included rich PK data from healthy subjects, as well as semirich and sparse data from MDR-TB patients from various parts of the world. To our knowledge, this is the largest set of PK data for cycloserine from TB patients analyzed using a nonlinear mixed-effects model. Weight, CrCL, and the presence or absence of the disease were identified as significant covariates on the PK parameters and explained some of the interindividual variabilities. Our simulations indicate that the current commonly used doses in practice for cycloserine, 250 to 500 mg once or twice daily, are not sufficient for MICs of >16 mg/liter (13). For higher MICs, a total daily dose of at least 1,500 mg is needed, which raises questions regarding its tolerability.

Recently, the WHO has regrouped drugs used in the treatment of MDR-TB (4). Cycloserine, along with four additional TB agents, is now recommended as part of the regimen for all MDR-TB patients, and hence more MDR-TB patients are expected to receive cycloserine. However, limited data are available on its PK/PD in TB patients. Several studies have reported the plasma concentrations of cycloserine, but many of them included 1 to 2 concentrations in therapeutic drug monitoring settings (5, 14–19). Although the typical *C*_max_ for cycloserine is usually thought to be between 20 to 35 mg/liter after a 250 or 500 mg dose in adults (20), a few studies have reported lower plasma concentrations in some MDR-TB patients using the same doses (16, 17). This typical range seems to be applicable to children as well. Kumar et al. recently reported an average plasma concentration of 32 mg/liter in children with MDR-TB after receiving an average dose of 14 mg/kg (∼500 mg dose), which is in accordance with early studies (21, 22).

In healthy Chinese volunteers, Zhou et al. have shown that cycloserine follows linear pharmacokinetics, with average *C*_max_ values of 19.4, 42.9, and 84.8 mg/liter after single doses of 250, 500, and 750 mg, respectively (23). In an earlier study by Zhu et al., they reported a lower *C*_max_ (14.8 mg/liter) for the 500 mg dose (24). It is worth noting that the average participant weights between the two studies were quite different (56 versus 78 kg), which could have contributed to the observed differences in the *C*_max_ value. In fact, our model showed that weight had a significant effect on *V*/*F*. Our population PK parameter estimates were comparable to what Zhu et al. have reported (24). This was not surprising, since we utilized those data in our model. On the other hand, Chang et al. reported lower absorption rate constant (*k_a_*) and *V*/*F* estimates (0.14 h^−1^ and 10.5 liters, respectively) using a one-compartment model with first-order absorption (25). This is possibly due to the differences in the structural models (including a lag time in our case) and the inclusion of covariates in our model (including weight as a covariate on *V*/*F*).

The significant effect of CrCL on CL/*F* was expected since cycloserine is approximately 70% renally cleared (26). The CL/*F* estimate in healthy subjects was about twice the CL/*F* estimate in patients. This could be due to the differences in the study settings. The healthy subjects had normal kidney and liver functions, fasted overnight, did not take other medications (except studied TB drugs), and were sampled extensively over 48 hours. In contrast, many of these variables were different or missing in the other included studies, which could have contributed to the observed difference between the two groups, given that, for example, food results in delayed absorption (24).

Cycloserine inhibits cell wall synthesis by targeting the formation of peptidoglycan, the same target as for beta-lactams but through a different mechanism of action (3, 27). Hence, the PD index for cycloserine was hypothesized to be *T*_>MIC_. Recently, Deshpande et al. confirmed that *T*_>MIC_ is indeed the efficacy driver for cycloserine in a hollow-fiber system model, indicating that a *T*_>MIC_ of 30% was associated with bactericidal activity, and 64% represented the 80% of the maximal kill (EC_80_) value (6). Both were included in our PK/PD analysis. The PTA increased significantly as the total daily dose increased. On the other hand, taking the total daily dose once per day versus dividing it did not affect the PK/PD breakpoint, with the exception of the 250/500-mg regimen, which had a higher PK/PD breakpoint than the 750 mg dose once daily. Interestingly, all of the 250-mg dosage regimens, including four times daily, failed to achieve a PK/PD breakpoint higher than 16 mg/liter. This suggests that our current dosing for cycloserine may not be sufficient for some strains, given the current tentative epidemiologic cutoff (ECOFF) value for cycloserine (between 32 and 64 mg/liter) (6, 28). Indeed, Yu et al. and Deshpande et al. have shown that approximately 30% and 26% of their tested isolates had MIC values higher than 16 mg/liter. For MIC of 32 mg/liter, a dose of at least 750 mg twice daily is needed, which is consistent with previous findings from Deshpande et al. (6). Therefore, we might need to identify patients who would most likely benefit from cycloserine based on their individual MICs. It is worth noting, however, that in general, cycloserine drug susceptibility testing is not performed in settings where MDR-TB is endemic.

The neuropsychiatric adverse events of cycloserine include anxiety, agitation, depression, psychosis, and, rarely, seizures (9, 10). Compared to other second-line agents, cycloserine has been associated with more frequent neuropsychiatric-related adverse events. A recent metaanalysis showed that the frequencies of psychiatric and central nervous system adverse events are 5.7 and 1.1%, respectively (29). These adverse events may be associated with elevated plasma concentrations of cycloserine (20). A few studies from the 1950s reported the use of a total daily dose of 1,000 to 1,500 mg. These showed mixed results in terms of the incidence of neuropsychiatric adverse effects, ranging from 6 to 77% (30–32); the latter included a very small sample size (*n* = 13). Holmes et al. indicated that the observed cases (*n* = 2) of psychiatric reactions had serum concentrations of >50 mg/liter, while cases (*n* = 2) with early symptoms of tremor, weakness, and mild disorientation had serum concentrations of >40 mg/liter (31). Hung et al. also reported a case with psychotic symptoms that had cycloserine plasma concentrations of >35 mg/liter (15). Further studies in this area are needed to define which PK parameter is associated with toxicity and whether increasing the exposure beyond the current recommended serum concentration range of 20 to 35 mg/liter is feasible from a safety perspective.

One of the limitations of our analysis is the inclusion of intensively sampled PK data for healthy subjects only. However, relatively large semirich data from two ongoing prospective studies in TB patients were also included. As the case with the nature of retrospective studies, these are prone to a potential inaccuracy in data collection; this can be another limitation for the retrospectively collected data (the fourth and fifth data sets). Also, the PD targets for *T*_>MIC_ were based on hollow-fiber studies evaluating the efficacy of cycloserine alone. Even though the treatment of MDR-TB includes at least 4 to 6 drugs given in combination, the current practice is to try to optimize each drug independently. At this time, true synergy for cycloserine with other TB drugs has not been proven. This is a limitation of our study. More importantly, although we have explored the *C*_max_ of the simulated regimens as a potential driver for toxicity, our analysis did not evaluate the safety of cycloserine *per se*. There are scant safety data for cycloserine, and we plan to examine that further in ongoing studies. Another limitation is that we had to assume the plasma protein binding of cycloserine to be zero, since it has not been reported in the literature.

In conclusion, cycloserine was best described by a one-compartment model with first-order absorption and a lag phase. Our simulations showed that dividing the dose minimally affects the PK/PD breakpoints, while resulting in a significant decrease in *C*_max_, which might reduce the neuropsychiatric adverse effects while preserving microbial killing. Target attainment analysis also showed that the current dosing of cycloserine is sufficient for MICs up to 16 mg/liter. Higher MICs require higher daily doses (>1,000 mg), the safety and tolerability of which need to be evaluated in future studies.

## MATERIALS AND METHODS

### Study data sets and subjects.

A total of five data sets were used for the analysis. The first was from healthy subjects (*n* = 12) recruited at the University of Arizona, and they were given 500 mg of cycloserine as a single dose on an empty stomach (24). They were intensively sampled over 48 hours at 0.25, 0.5, 0.75, 1, 1.5, 2, 2.5, 3, 4, 6, 8, 10, 12, 14, 24, 36, and 48 hours post dose. The second data set represents patients with MDR-TB from Tbilisi, Georgia (*n* = 69) who were given 250 to 1,000 mg of cycloserine. They were enrolled in a prospective observational study, and samples were mainly collected at 0, 2, 6 to 8, 10 to 12, and 24 hours approximately 4 to 6 weeks after initiating treatment. The third data set represents MDR-TB patients from Bangladesh (*n* = 42) given 500 to 1,000 mg of cycloserine. They were enrolled in a multicountry, prospective, observational study that collected blood samples at 1, 2, 6, and 12 hours 2 weeks after treatment initiation. Blood samples also were collected at 2 and 6 hours after 4 and 8 weeks following treatment initiation. The fourth data set comes from patients (*n* = 54) with MDR-TB or nontuberculous mycobacteria from National Jewish Health (NJH) in Denver, CO. This included sparse clinical samples (1 to 2 samples per patient), mainly at 2 and 10 hours post dose. Finally, the fifth data set also included sparse clinical samples (mainly at 2 and 6 hours) from a retrospective study involving three TB centers in the United States (*n* = 70), A. G. Holley Hospital (AGH) in Florida, the Texas Center for Infectious Diseases (TCID), and the University of Texas Health Science Center at Tyler (UTHSCT). The cycloserine dose for the fourth and fifth data sets ranged from 250 to 750 mg.

The institutional review boards (IRBs) of all participating sites reviewed and approved the studies included in this analysis (AGH: Florida IRB 2014-12; Emory University: IRB 00083639; International Centre for Diarrhoeal Diseases Research, Bangladesh (icddr,b): IRB PR-15121; National Center for TB and Lung Diseases (NCTLD): IRB 00007705; NJH: IRB HS-827; TCID: IRB 14-013; University of Florida: IRB 201300638; University of Virginia: IRB 18452; and UTHSCT: IRB 09 to 016). For the prospective studies, written informed consent was obtained from all participants or their legal guardians. For the retrospective studies, informed consent was waived by the respective IRBs. The research was performed in accordance with the Declaration of Helsinki and institutional standards.

### Drug quantification.

Cycloserine plasma concentrations for healthy subjects were measured using a validated high-performance capillary electrophoresis assay, as described by Zhu et al. (24), which also was used for the NJH data. Blood samples from the Georgia and Bangladesh studies were centrifuged, and plasma samples were stored at −80°C until assayed. Total plasma concentrations for both studies were measured using a validated liquid chromatography tandem mass spectrometry assay, performed at the Infectious Disease Pharmacokinetics Laboratory at the University of Florida. The analysis was performed on Thermo Scientific TSQ Endura or TSQ Quantum Ultra mass spectrometers. The curve was linear over the range of 1.25 to 50 mg/liter. Samples with concentrations that exceeded 50 mg/liter were diluted and reanalyzed with similarly diluted quality control samples. The coefficients of variation of validation quality control samples were 4.7 to 8.2% for intraday precision and 4.2 to 6.3% for interday precision. The intraday and interday accuracy ranges were 97.4 to 110.6 and 95.7 and 107.0%, respectively. The cycloserine concentrations for patients from the U.S. sites were collected from their patient charts. These samples were assayed in C. A. Peloquin’s laboratory. From 1988 to 2009, that was located at the NJH in Denver. From 2009 onward, that was located at the College of Pharmacy, University of Florida.

### Population pharmacokinetic modeling and Monte Carlo simulations.

Monolix (2018R1) was used to build the population PK model. One- and two-compartment models, using first- and zero-order elimination, were used to fit the data. Interindividual (ω) and interoccasion variabilities (γ) were also estimated, assuming log-normal distribution. The tested residual error models included additive, proportional, and combined error models. The intensively sampled PK data from healthy subjects were used first to build and assess the structural model. After establishing that, the other semirich and sparse PK data were added. The ratio of eigenvalues was utilized in assessing the correlation and overparameterization of the population parameters. Age, sex, body weight, body mass index, absence or presence of disease (i.e., healthy subjects versus patients), type of disease (i.e., DS-TB, MDR-TB, pre-XDR-TB, and XDR-TB), CrCL, and site also were tested as covariates on the PK parameters. CrCL was calculated using the Cockcroft-Gault equation. HIV status was not available for many patients, and hence it was not considered in our model. After building the structural model, covariates were added in a stepwise fashion, with the most significant covariate being entered first, using a forward inclusion approach. A *P* value of <0.05 was considered statistically significant. An exponential model (equation 1) was used for categorical variables. For continuous variables, a power function was used after normalizing the individual value to the median (equation 2).

(1) $$\mathrm{CL}={\mathrm{CL}}_{\mathrm{POP}}\cdot \left[\mathrm{if}\;\mathrm{sex}=\mathrm{male},\;{\;e}^{\beta_{\mathrm{male}}}\right]$$

(2) $$\mathrm{CL}={\mathrm{CL}}_{\mathrm{POP}}\cdot {\left(\frac{\mathrm{age}}{{\mathrm{age}}_{\mathrm{median}}}\right)}^{\beta_{\mathrm{age}}}$$

CL_POP_ is the population value of clearance (CL), and β is the estimated effect of sex or age on CL.

The structural model and addition of covariates were evaluated using the log-likelihood ratio (Δ−2LL ≥ 3.84 for 1 degree of freedom), goodness-of-fit plots, and the physiological plausibility of the model parameter estimates. VPC was used to evaluate and validate the final model by simulating cycloserine concentrations for 500 patients using the original data set and the final model.

The final PK estimates were used in the mlxR package (v3.3.0) in R software to simulate the time course of cycloserine concentrations over 24 hours. For each dosage regimen, we simulated the concentrations every 0.2 hour for 1,000 TB patients at steady state. Demographic data for the simulated patients were randomly sampled, assuming they were normally distributed, using the mean values and standard deviations from the original data set. A correlation of 0.4 also was taken into account when simulating weight and CrCL; the value was obtained from the observed correlation in our data set. We simulated three doses, 250, 500 and 750 mg, with different frequencies (once, twice, three times, and four times daily). For the 750-mg dose, we did not simulate the four times daily regimen, since it would result in a high total daily dose (i.e., 3,000 mg) that most patients would likely not tolerate. Instead, we simulated 250 mg in the morning and 500 mg in the evening, a dosage regimen that is commonly seen in clinical practice. We used PK/PD targets for *T*_>MIC_ values of ≥30% and ≥64%, representing bactericidal activity and 80% of the maximal kill (EC_80_), respectively (6). We assumed that cycloserine does not bind to plasma proteins, as there are no data on its protein binding. The studied range of MIC values was 4 to 64 mg/liter, based on the MIC distribution reported in the literature (5, 6). Using R software, the PTA was calculated as the fraction of simulated patients achieving the PK/PD target at each MIC for each regimen. We selected a PTA of at least 90% for the highest MIC as the PK/PD breakpoint.

## Supplementary Material

Supplemental file 1
