# Supplementary material for: Cycloserine Population Pharmacokinetics and Pharmacodynamics in Patients with Tuberculosis
Source: Antimicrob Agents Chemother. 2019 Apr 25;63(5):e00055-19. doi: 10.1128/AAC.00055-19 (PMC6496076; doi:10.1128/AAC.00055-19)
Supplement: Supplemental file 1 [file AAC.00055-19-s0001.pdf]

SUPPLEMENTARY MATERIALS

Figure S1. Goodness-of-fit plots for the final model.

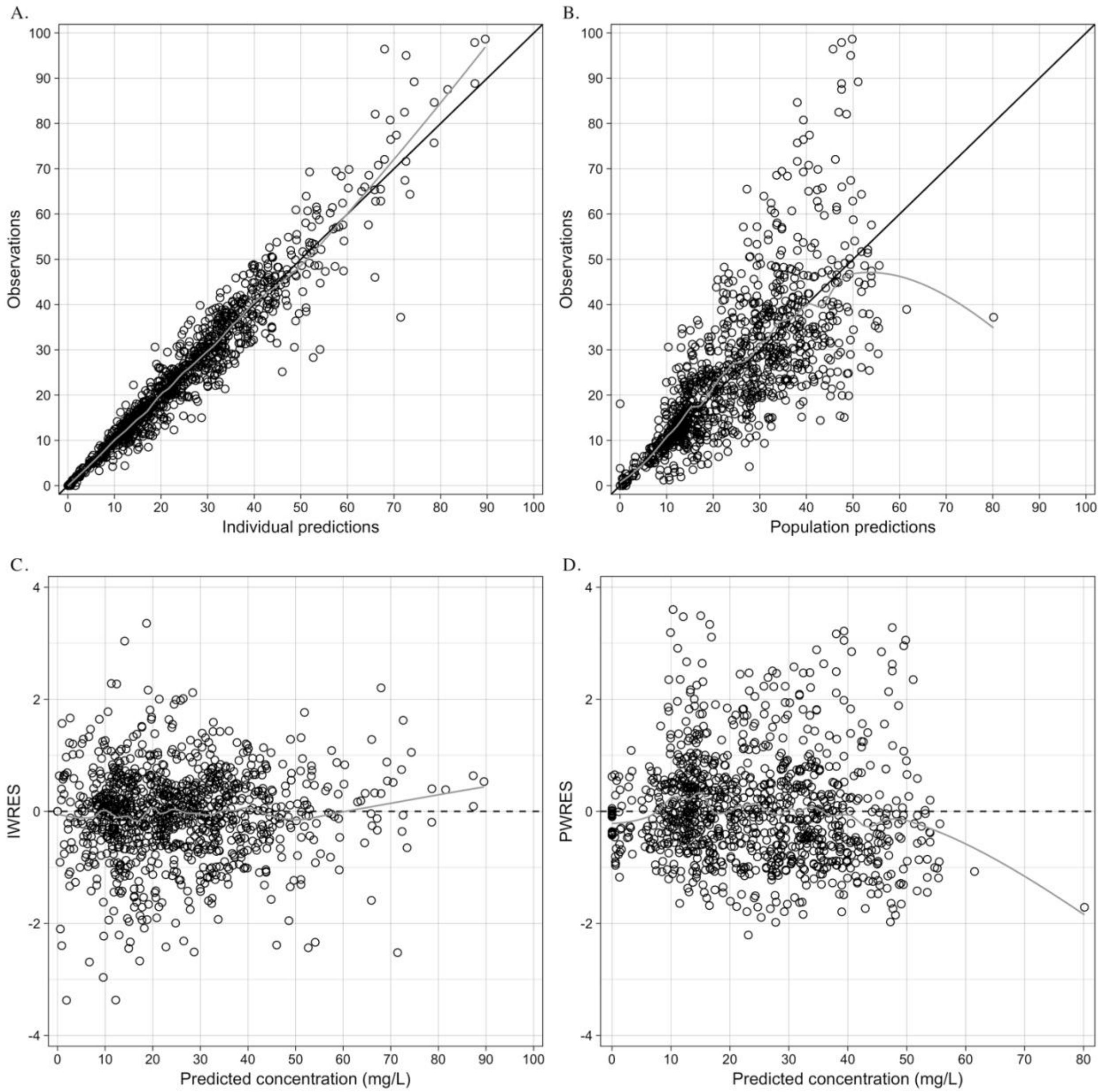

A) Observations versus individual predictions plots. B) Observations versus population predictions plots. C) Individual weighted residuals (IWRES) versus concentrations; five observations had values >4 or <4 and were not plotted. D) Population weighted residuals (PWRES) versus concentrations.

Figure S2. Visual predictive checks stratified by the given dose.

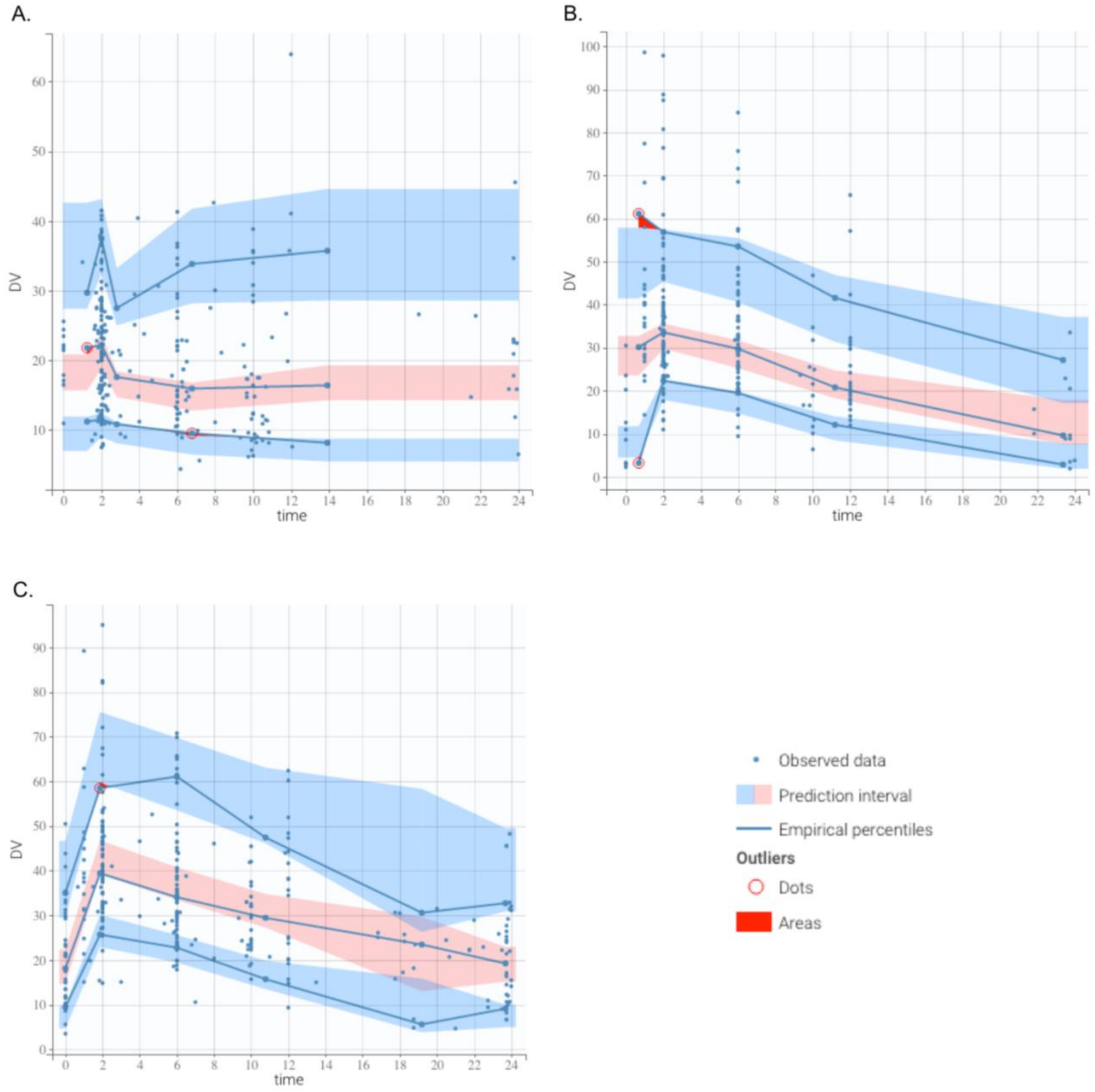

VPC stratified by the given dose. A) VPC for all the 250 mg doses, including once daily, twice daily (every 12 hours), and irregular twice daily dosing (i.e. dose given at time 0 and 6 or 8 hours for example). B) VPC for the 500 mg doses. C) VPC for the 750 mg doses.
